# Urothelial Papilloma of the Urinary Bladder in Children: Report of Two Cases

**DOI:** 10.1055/s-0040-1705154

**Published:** 2020-04-23

**Authors:** Kata Davidovics, Sandor Davidovics, Andras Farkas, Noemi Benedek, Tamas Tornoczki, Daniel Kardos, Anna Davidovics, Peter Vajda

**Affiliations:** 1 Department of Pediatrics, University of Pecs, Pecs, Hungary; 2 Department of Pathology, University of Pecs, Pecs, Hungary; 3 Languages for Specific Purposes, University of Pecs Medical School, Pecs, Hungary

**Keywords:** urinary bladder, neoplasm, tumor, children

## Abstract

Urothelial neoplasms of the bladder (UNB) are considerably rare throughout the pediatric population. UNB develops from the urothelial tissue in the form of a benign disease, generally favoring a successful prognosis in the majority of cases. The authors present the diagnosis and treatment regarding two medical case reports in which urothelial papilloma was diagnosed and effectively treated. **Case 1** : A 15-year-old male patient was presented to our clinic complaining of a painless yet distinctive, macroscopic form of hematuria. Following a routine examination, which included ultrasound (US) and intravenous pyelography, the urethrocystoscopy revealed an intravesical solitary lesion positioned in the vicinity of the left ureteral orifice. Additionally, histology confirmed urothelial papilloma. During the follow-up, laboratory, urinary control tests, and US results all proved negative. **Case 2** : A 13-year-old male patient was admitted to our clinic and examined, in regard to complaints associated with recurrent abdominal pain. The pathology was discovered incidentally on abdominal US. Preoperative US and magnetic resonance imaging (MRI) studies ensued, resulting in a scheduled MRI, followed by urethrocystoscopy, which confirmed an intravesical solitary lesion positioned near the right ureteral orifice. Histology revealed urothelial papilloma. During the follow-up control cystoscopy, one resection was repeated due to the presence of a residual tumor. Today, 10 years since the presence of uroepithelial papilloma, both patients are asymptomatic and tumor-free. If there is likely suspicion of recurrence, cystoscopy is recommended.

## Introduction

While more frequently seen in an adult population, epithelial (urothelial) tumors of the urinary bladder are among the top 10 most common types of neoplasms, including a significant potential regarding malignancy and a dismal prognosis. Distinctly, urothelial neoplasm of the bladder (UNB) is extremely rare among the pediatric population (below 18 years of age).

Rhabdomyosarcoma is the most common tumor associated with the urinary bladder among children. Rhabdomyosarcoma, which is malignant, develops in the mesenchymal tissue, and generally features a bleak prognosis.

In contrast, UNB (urothelial papilloma or UP, inverted urothelial papilloma, or IUP, papillary urothelial neoplasm, associated with a low malignant potential, or PUNLMP, a low-grade urothelial carcinoma, or LGUC, and high-grade urothelial carcinoma—HGUC) arises from the epithelial tissue, and except in consideration of HGUC, these tumors generally result in a favorable prognosis. 1 Interestingly, only a few papers regarding UNB among the pediatric or adolescent population can be found throughout English literature.

Among these, only 11 cases were found, in which UP was prevalent during the diagnosis. 2 3 4

The authors present two cases in reference to urothelial papilloma, diagnosed and treated in their respective department from 2007 through 2017.

## Case Reports

Data regarding two patients were retrospectively analyzed, including the symptoms, imaging methods, local extension of the lesion, histological evaluation, treatment, and follow-up.

### Case 1

In 2007 a 15-year-old-male was presented with macroscopic hematuria and dysuria, without signs of systemic infection, to an external medical institute. His family history proved negative for any type of urogenital diseases or tumors including risk factors (smoking, alcohol, and promiscuity).

Laboratory tests were negative and kidney function and inflammatory markers were within the normal range. The urine analysis test detected significant (>10 ^5^ cfu/mL) *Escherichia coli* bacteriuria, which was treated according to its antibiotic sensitivity, for a span over 2 weeks. Shortly thereafter, an abdominal ultrasound (US) examination revealed an intraluminal lesion in the bladder.

Following antibiotic therapy, the patient's symptoms did not reoccur. Additional diagnostic equipment regarding additional forms of treatment was achieved at the author's institute to clarify the lesion.

During further diagnostic workup via US, on the repeated US a 15 × 15 mm, hyper reflective, solid, solitary lesion was detected in the urinary bladder adjacent to the orifice of the left ureter. Intravenous pyelography (IVP) was made and no obstruction was detected. On the fifth day following admission, the urethrocystoscopy was performed and the papillary tumor of the bladder was endoscopically resected.

In regard to the resection, a 11.5 Fr resectoscope was used. The removal was made by the use of diathermy. The histological examination revealed urothelial papilloma (R0 resection). The follow-up included laboratory and urine tests, US examinations at 3-month intervals, all within the first 3 years, then once a year thereafter.

**Fig. 1:**
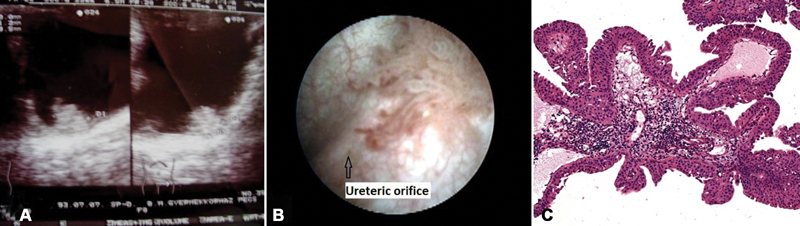
On ultrasound scan, a 15 × 15 mm, solid, hyperreflective, solitary lesion was detected in the urinary bladder next to the orifice of the left ureter. ( **A** ) During urethrocystoscopy, a typical uroepithelial papilloma was detected next to the left ureteral orifice. ( **B** ). Histological finding was uroepithelial papilloma. ( **C** ) Case 1.

Distinctly, no further abnormalities were detected. At the time, a cystoscopical follow-up was not routinely indicated. The patient is now 25 years old, asymptomatic, and tumor free, for the past 10 years ( Fig. 1 ).

### Case 2

In 2017, a 13-year-old-male was presented suffering from recurrent abdominal pain of unknown origin. His family history proved positive for maternal Crohn's and celiac disease; however, all risk factors proved negative.

Laboratory and urine tests were negative. The US showed an 18 × 20 × 21 mm large, lobulated, hyperreflective lesion adjacent to the orifice of the right ureter. A magnetic resonance imaging (MRI) examination was made to clarify the extent of the neoplasm. It featured a local lesion without any sign of infiltration. Urethrocystoscopy revealed a papillary tumor in the vicinity of the bladder, which was resected with the use of a diathermy, through a 11.5 Fr resectoscope. The histological examination revealed urothelial papilloma (R1 resection).

However, control US and urine test results proved negative, a control cystoscopy was performed 1 month following the incomplete (R1) resection. During this intervention, a residual tumor was discovered at the same location as the primer lesion. A re-resection was endoscopically performed through the same means as previously achieved.

**Fig. 2:**
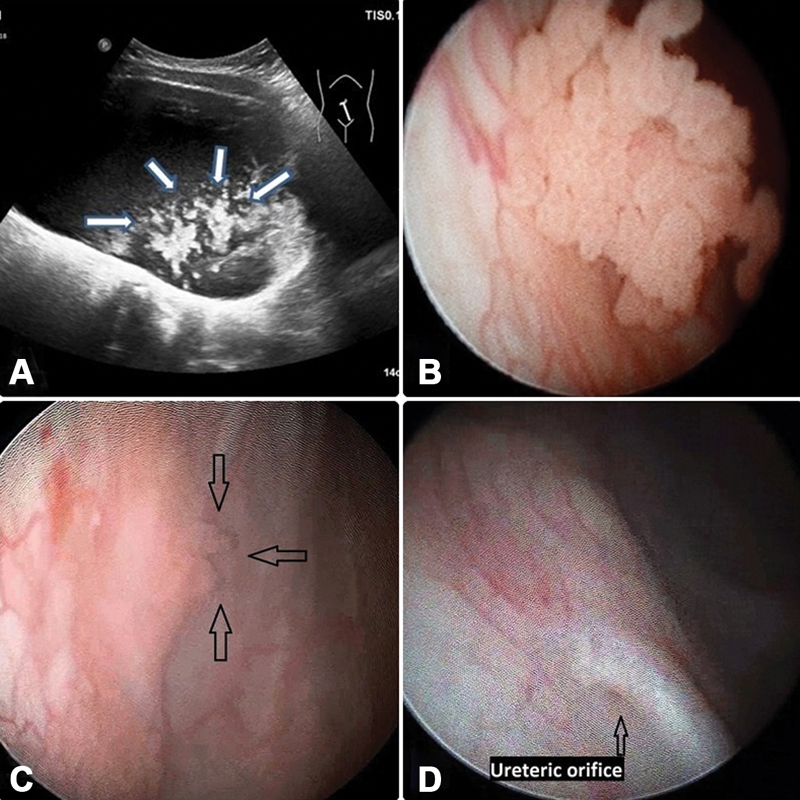
Next to the orifice of the right ureter, an 18 × 20 × 21 mm large, lobulated, and hyperreflective lesion was detected on ultrasound. ( **A** ) Urethrocystoscopy revealed a classical uroepithelial papilloma next to the right ureteral orifice. ( **B** ) One month after surgery, the control cystoscopy indicated residual papilloma. ( **C** ) In the third postoperative month, the control cystoscopy revealed no residual lesion. ( **D** ) Case 2.

The histology discovered a residual urothelial papilloma (R0 resection). Three months following the re-resection, control laboratory and urine tests, dipstick, and US all proved negative. A repeated cystoscopy procedure showed no residual or recurrent tumor in the bladder. The patient is now 14 years old. He is asymptomatic and has been tumor free for the past years ( Fig. 2 ).

## Discussion

Epithelial tumors associated with the bladder are considered among the top 10 most commonly seen neoplasms throughout the adult population.

Notably, these neoplasms are considerably rare among the pediatric age group.

According to WHO 2016 classification of tumors associated with the urinary bladder, UNB (carcinoma in situ, LGUC, HGUC, PUNLMP, UP, IUP, and urothelial hyperplasia and dysplasia) is, indeed, an extremely rare condition among those within the pediatric age group. 1

Startlingly, very few publications can be found in English literature, in reference to UNB in the pediatric population. 2 3 4 5 6 7

Fine et al analyzed surgical and histological data regarding three pediatric urology units' cases, all under the age of 20 years, and afflicted with various forms of UNB. Twenty-three cases were reviewed, and urothelial papilloma were prevalent in only two cases. 2

Berrettini et al retrospectively analyzed the data regarding three tertiary pediatric urology units with the diagnosis of UNB from 1999 through 2013. In consideration of the eighteen cases associated with UNB, only eight patients were diagnosed with urothelial papilloma. 3

Based on these studies, gross painless hematuria appears to be the most common presenting symptom regarding UNB (nearly 80% of the cases). 3

Cystoscopy and transurethral resection of the lesion appear to be a successful form of treatment in the majority of cases. 4

Although pediatric guidelines are not available regarding the management of UNB, US may bear sufficient sensitivity toward effectively establishing the clinical diagnosis. However, the endoscopy has the highest level of sensitivity and specificity, an additional MRI or CT scan may prove benefit toward clarifying the malignant potential of the detected lesion. 5

In 2007, we made IVP routinely to visualize if there is any likelihood of an obstruction or intraluminal lesion in the bladder; however, today, this diagnostic tool is rarely used. In cases due to the low incidence of papillomas, there is, as of yet, no standardized guidance regarding the follow-up.

Studies focused on the adult population highlighted the recurrence of papillomas in several cases; therefore, US is advocated as a form of follow-up. 6 To perform routine urethrocystoscopy in the form of a follow-up, procedure remains a controversial issue, since the data in reference to the literature (low incidence) is considerably limited, and, as a result, the exact recurrence rate regarding these tumors cannot be identified. However, histologically, our cases were not PUNLMPs; therefore, based on the study of Di Carlo et al, the follow-up is often times similar among children with UP. 7

If there is any suspicion of recurrence or residual disease (microscopic or macroscopic hematuria without infection, US finding), it is highly recommended to perform cystoscopy, which the author encourage enthusiastically.

## Conclusions

Due to the low incidence of UNB among children, there is no specific pediatric guideline in reference to an effective form of treatment or follow-up. Complete transurethral resection of uroepithelial papilloma may likely provide suitable levels regarding minimally invasive and successful treatment during childhood; however, a urinary dipstick for hematuria and US scan may indeed prove beneficial in the follow-up procedure toward detecting residual and/or recurrent forms of pathology.

According to our limited experience, cystoscopy is highly recommended in the early postoperative period to detect incomplete resection of the tumor, and considered as an optional form of treatment during long-term follow-up. However, if and when there is any suspicion of the presence of a tumor, a cystoscopy is deemed absolutely necessary to perform. 8

## Notes

New Insights and the Importance for the Pediatric Surgeon Authors present two cases of uroepithelial neoplasm of the bladder, in which uroepithelial papilloma was diagnosed and treated in childhood. The therapy is mainly individual in this very rare entity, because there is no specific pediatric guideline for the treatment or follow-up in pediatric population.
